# Bright tongue sign as a radiological clue of bulbar onset amyotrophic lateral sclerosis: A case report

**DOI:** 10.1016/j.radcr.2025.05.044

**Published:** 2025-06-12

**Authors:** Seid Mohammed Shobe, Dereje Melka, Mesfin Mulugeta, Leul Adane

**Affiliations:** a Department of Radiology, Addis Ababa University School of Medicine, Addis Ababa, Ethiopia; b Department of Neurology, Addis Ababa University School of Medicine, Addis Ababa, Ethiopia

**Keywords:** Bright tongue sign, Case report, MRI

## Abstract

Amyotrophic lateral sclerosis (ALS) is a fatal neurodegenerative disease characterized by degeneration of motor neurons, with the tongue often involved in clinical presentation. In this case, a 60-year-old female presented with progressive choking episodes and speech slurring over 9 months, exhibiting dysarthria, prominent tongue atrophy, fasciculations, and hyperreflexia. Needle electromyography (EMG) showed diffuse chronic neurogenic changes with signs of active denervation changes prominent on the tongue and right arm with normal sensory nerve studies. Magnetic resonance imaging (MRI) brain imaging revealed a Diffuse T1 Weighted image (T1WI) hyperintense of tongue known as "bright tongue sign" indicating fatty infiltration of tongue muscles, consistent with neurogenic atrophy. This case underscores the importance of recognizing this characteristic tongue hyperintensity as a valuable radiological clue in diagnosing bulbar-onset ALS and highlights the potential for early diagnosis to improve patient management and outcomes

## Introduction

Amyotrophic lateral sclerosis (ALS) is a progressive neurodegenerative disease characterized by the degeneration of both upper and lower motor neurons [1]. This degeneration leads to atrophy of voluntary skeletal muscles and ultimately results in paralysis [2]. The disease typically begins in the limb or bulbar muscles and progresses to contiguous regions, eventually involving the respiratory muscles [2].

Early diagnosis of ALS is essential for timely intervention, effective symptom management, and enrollment in clinical trials [3]. However, the diagnosis is primarily clinical and is often delayed due to the absence of specific biomarkers [4,5]. In this context, imaging findings can provide valuable diagnostic support by offering noninvasive clues that complement clinical evaluation [6].

Magnetic resonance imaging (MRI) of the brain is frequently used in the evaluation of neurological disorders. The tongue is often visible on sagittal brain MRI, and its appearance may provide important diagnostic information. However, abnormalities of the tongue are frequently overlooked by both radiologists and neurologists [7]. A hyperintense signal of the tongue on T1-weighted imaging (T1WI), referred to as the “bright tongue sign,” is a radiological clue suggestive of fatty infiltration of the tongue musculature [[7], [8], [9], [10]].

This sign is most commonly associated with ALS cases involving bulbar muscles. However, it is not pathognomonic and may also be observed in other neuromuscular disorders with tongue and/or bulbar involvement, such as late-onset Pompe disease, myotonic dystrophy, and Kennedy disease [7,8].

Here, we present a case of ALS with characteristic MRI findings of tongue hyperintensity on T1WI and emphasize its potential value as a supportive diagnostic marker.

## Case presentation

The patient was a 60-year-old woman with no known personal or family history of neurological or neurodegenerative disorders. She sought medical attention on October 10, 2023, due to a 9-month history of progressively worsening episodes of choking while swallowing and slurred speech. She initially reported difficulty articulating words and occasional choking on liquids, symptoms that gradually increased in frequency and severity. Her medical history was unremarkable, with no prior surgeries, trauma, or chronic illnesses. She denied any history of weight loss, fever, night sweats, or recent infections.

On physical examination, her vital signs were within normal limits, and a general systemic examination was unremarkable. Neurological assessment revealed intact cognitive function and normal consciousness. Cranial nerve examination showed dysarthria, prominent tongue atrophy, fasciculations, and normal facial movement. She exhibited exaggerated reflexes in both knees, and fasciculations were observed in the right arm. Muscle strength, tone, and bulk in the limbs were normal, and sensation was intact. No other motor deficits were detected.

Laboratory investigations—including a complete blood count, renal and liver function tests, serum electrolytes, and inflammatory markers—were all within normal limits. Electromyography (EMG) demonstrated diffuse chronic neurogenic changes with active denervation, most prominently affecting the tongue and right arm muscles, supporting a diagnosis of motor neuron disease.

**Fig. 1:**
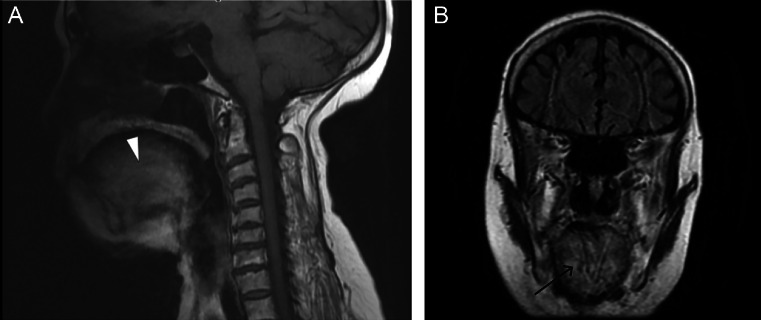
(A) Mid-sagittal T1WI brain MRI demonstrates diffuse hyperintense signal within the tongue musculature (White arrowhead). (B) Coronal FLAIR brain MRI at the level of the mid-tongue shows diffuse, symmetrical hyperintense signal within the intrinsic tongue muscles. (Black arrow).

Mid-sagittal brain MRI demonstrated diffuse T1WI within the tongue musculature, consistent with fatty infiltration the bright tongue sign (Fig. 1A). Coronal FLAIR images at the level of the tongue showed diffuse, symmetrical hyperintense signal within the intrinsic tongue muscles, clearly delineating bilateral involvement (Fig. 1B). Integration of these imaging findings with clinical assessment and electrophysiological studies supported the diagnosis of bulbar-onset ALS.

Management included initiation of riluzole therapy to slow disease progression and referral to speech therapy to address dysarthria. Nutritional support was discussed, including counseling on percutaneous gastrostomy tube insertion, although the patient declined the procedure at that time. She was educated about the disease prognosis, supportive care options, and the importance of regular follow-up.

During subsequent follow-up visits, the patient’s condition remained relatively stable with continued speech therapy and medical management. She did not develop significant respiratory compromise during the initial observation period but was counseled on recognizing signs of respiratory decline and the need for future supportive interventions.

## Discussion

Brain MRI is frequently ordered in patients with suspected neurological conditions. Although the tongue is often incidentally included in the imaging field, it offers a valuable yet frequently overlooked diagnostic opportunity for detecting neuromuscular disorders.

The bright tongue sign on brain MRI has emerged as a potential radiological indicator for a range of neuromuscular diseases affecting the bulbar or tongue musculature, including ALS, late-onset Pompe disease, myotonic dystrophy, and Kennedy disease [7,8]. This sign is characterized by diffuse, symmetrical hyperintensity of the tongue muscles on T1WI, reflecting neurogenic atrophy with subsequent fatty replacement of the tongue musculature.

MRI-detected structural abnormalities of the tongue are observed in more than half of ALS patients (24 out of 43) and are associated with increased disease severity and a longer disease course, averaging 18 months compared to 10 months in patients without these abnormalities [9]. The authors suggest that tongue MRI may serve as a useful biomarker for lower motor neuron involvement and disease progression, providing valuable prognostic information beyond clinical assessments [9]. Furthermore, the presence of tongue abnormalities correlates with lower functional scores, underscoring their potential role in evaluating disease severity in ALS [9].

In a study evaluating sagittal T1WI brain MRIs of patients with various neuromuscular disorders, one-third of cases demonstrated tongue hyperintensity on T1WI [7]. This feature is often overlooked or underreported in routine MRI evaluations, yet it may serve as an important diagnostic clue, particularly in cases of respiratory failure of unclear etiology [7]. The study further suggests that recognizing tongue involvement on brain MRI can aid in the early diagnosis of Pompe disease, although these abnormalities are not specific to late-onset Pompe disease and may also be present in other neuromuscular disorders [7] . The findings highlight the potential value of paying closer attention to the tongue during MRI review, which may improve diagnostic accuracy for neuromuscular conditions [7].

A study assessing brain MRI in patients with suspected ALS demonstrated that a significant proportion of diagnosed ALS cases (45 out of 62), particularly those with bulbar and flail-arm subtypes, exhibited this characteristic tongue hyperintensity. Additionally, 88% of these patients experienced dysphagia, predominantly during the oral phase. [10]. Interestingly, some non-ALS patients also showed this imaging feature but did not experience dysphagia. Additionally, ALS patients with tongue hyperintensity on MRI had lower vital capacities, suggesting that this MRI feature might be associated with a poorer prognosis [10]. Overall, this radiological marker appears to be linked to disease severity and respiratory decline in ALS [10].

Although not pathognomonic, the imaging feature of tongue hyperintensity on T1WI is a valuable adjunct in the diagnosis of ALS, particularly when interpreted alongside clinical and electrophysiological findings. Its diagnostic utility, however, warrants further validation through larger, well-designed cohort studies to better define its sensitivity and specificity.

Greater awareness of this underrecognized MRI finding among healthcare professionals may facilitate earlier detection of ALS. As neuroimaging techniques continue to evolve, integrating this diagnostic imaging feature into diagnostic protocols may enhance early identification and differential diagnosis of ALS, potentially leading to more timely interventions and improved patient outcomes.

## Conclusion

In conclusion, this case underscores the significance of recognizing tongue hyperintensity on MRI as an indirect imaging marker of bulbar-onset ALS. Early identification of this radiological feature can aid clinicians in improving diagnostic accuracy and facilitating timely, targeted management strategies for patients with suspected ALS.

## Declaration of generative AI and AI-assisted technologies in the writing process

During the preparation of this work, the author(s) used ChatGPT (OpenAI) to assist with language refinement and manuscript editing. After using this tool, the author(s) carefully reviewed and edited the content as needed and take(s) full responsibility for the final content of the publication.

## Patient consent

The authors confirm that written informed consent was obtained from the patient for the publication of this case report in Radiology Case Reports.
